# Supplementary material for: Printable magnesium ion quasi-solid-state asymmetric supercapacitors for flexible solar-charging integrated units
Source: Nat Commun. 2019 Oct 29;10:4913. doi: 10.1038/s41467-019-12900-4 (PMC6820525; doi:10.1038/s41467-019-12900-4)
Supplement: Supplementary file 1 — Supplementary information [file 41467_2019_12900_MOESM1_ESM.pdf]

Supplementary Information

Printable Mg-ion quasi-solid-state asymmetric supercapacitors for flexible solar-charging integrated units

Zhengnan Tian,[1†] Xiaoling Tong,[1†] Guan Sheng,[2] Yuanlong Shao,[2*] Lianghao Yu,[1] Vincent Tung,[2] Jingyu Sun,[1,3*] Richard B. Kaner,[4*] and Zhongfan Liu[1,3,5]

[1]*College of Energy, Soochow Institute for Energy and Materials InnovationS (SIEMIS), Jiangsu Provincial Key Laboratory for Advanced Carbon Materials and Wearable Energy Technologies, Soochow University, Suzhou 215006, P. R. China.*
[2]*College Physical Sciences and Engineering Division, King Abdullah University of Science and Technology, Thuwal 23955-6900, Saudi Arabia.*
[3]*Beijing Graphene Institute (BGI), Beijing 100095, P. R. China.*
[4]*Department of Chemistry and Biochemistry, Department of Materials Science and Engineering, and California NanoSystems Institute, University of California, Los Angeles (UCLA), USA.*
[5]*Center for Nanochemistry (CNC), College of Chemistry and Molecular Engineering, Peking University, Beijing 100871, P. R. China.*

*Corresponding author. E-mail: sunjy86@suda.edu.cn (J.Y.S.); yuanlong.shao@gmail.com (Y.L.S.); kaner@chem.ucla.edu (R.B.K.)

†These authors contributed equally to this work.

Supplementary Figures

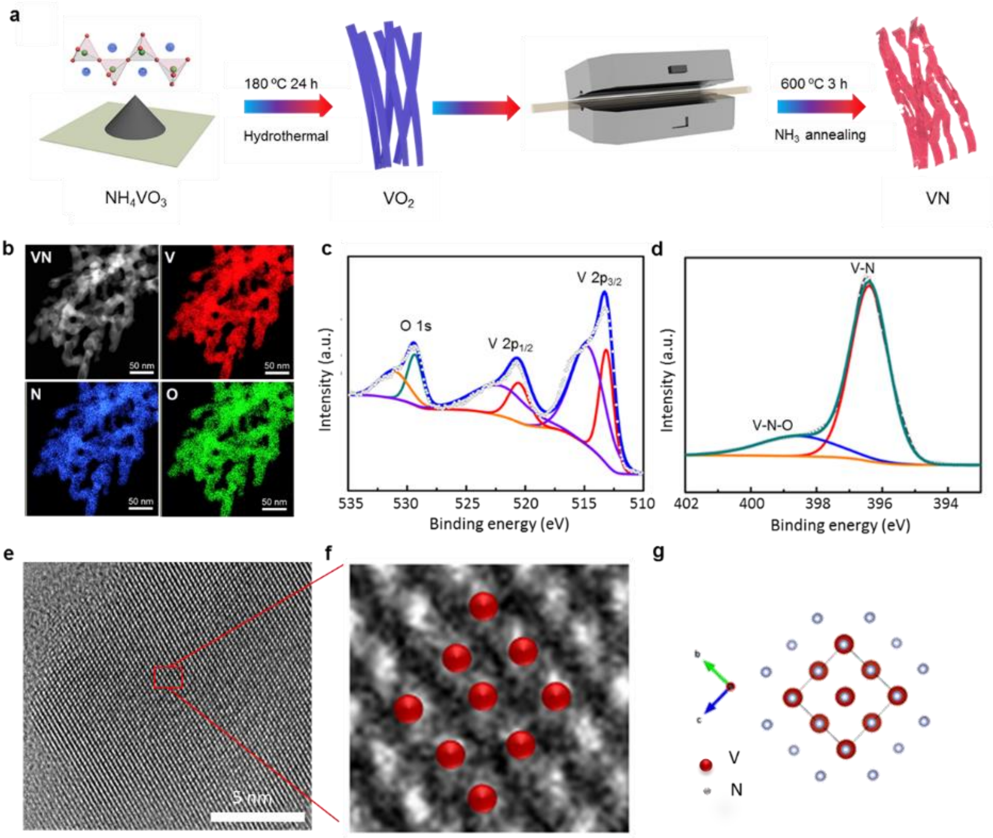

Supplementary Figure 1. Synthesis and characterization of VN. (a) Schematic illustration of the synthetic process of VN. (b) Elemental mapping of N, V and O in the as-synthesized VN material. (c) High-resolution XPS V 2p spectrum of VN. (d) High-resolution XPS N 1s spectrum of VN. (e, f) STEM images of VN. (g) Crystal unit cell presenting the (200) plane of VN.

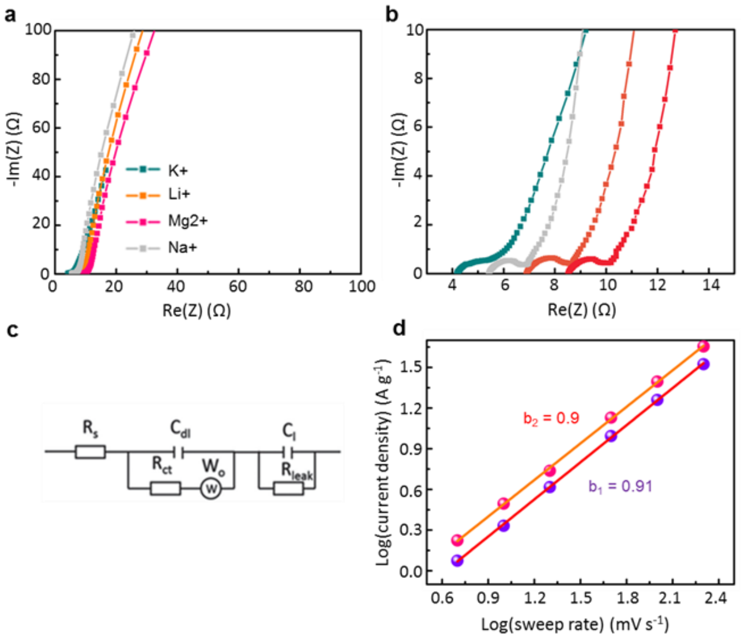

Supplementary Figure 2. EIS spectra in different electrolytes and *b*-value determination of VN. (a) EIS spectra of VN electrode in different electrolytes. (b) Detailed high-frequency region corresponding to (a). c) A Randles equivalent circuit. The equivalent circuit is used to fit the Nyquist curves. d) Calculated *b*-value of the anodic peak and cathodic peak.

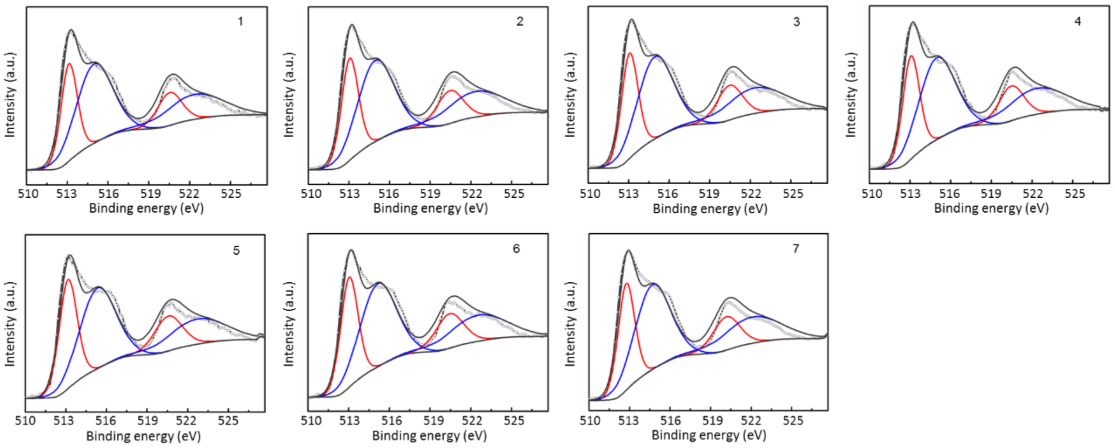

Supplementary Figure 3. ***Ex situ*** **XPS V 2p spectra.**

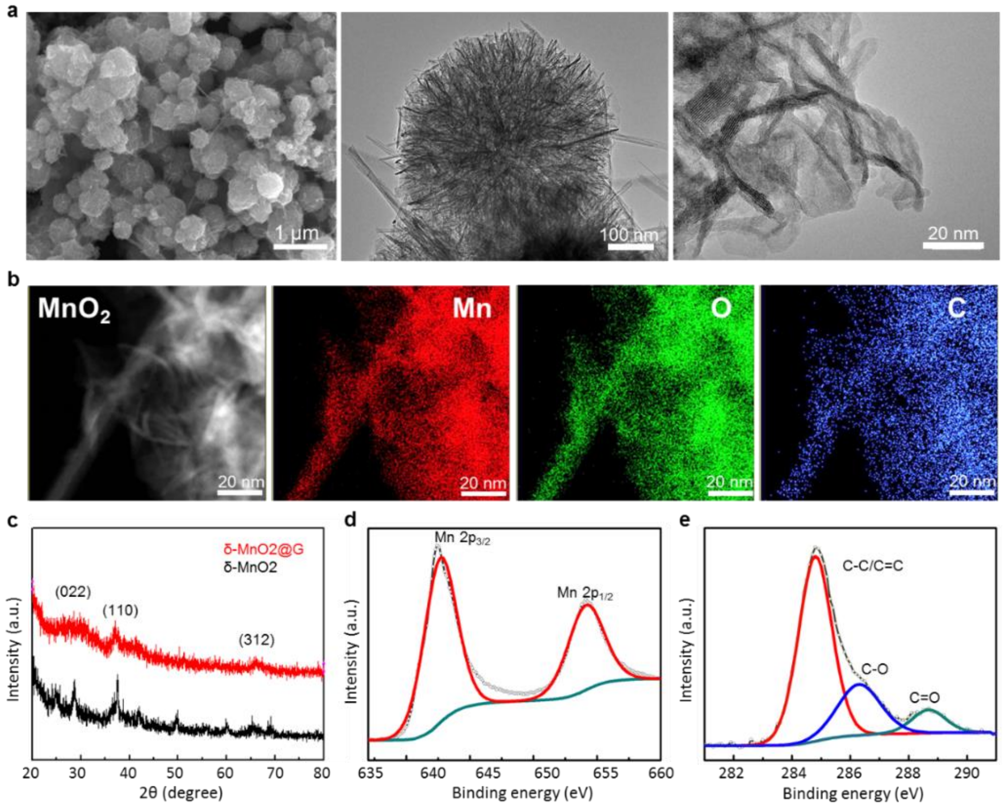

Supplementary Figure 4. Characterization of as-prepared MnO_2@C. (a) SEM (left panel) and TEM (middle and right panel) images of obtained MnO_2@C composite. (b) Elemental mapping of Mn, O and C in MnO_2@C composite. (c) XRD patterns of the MnO_2@C composite and bare MnO_2. (d, e) High-resolution XPS Mn 2p (d) and C 1s (e) spectra of as-prepared MnO_2@C composite.

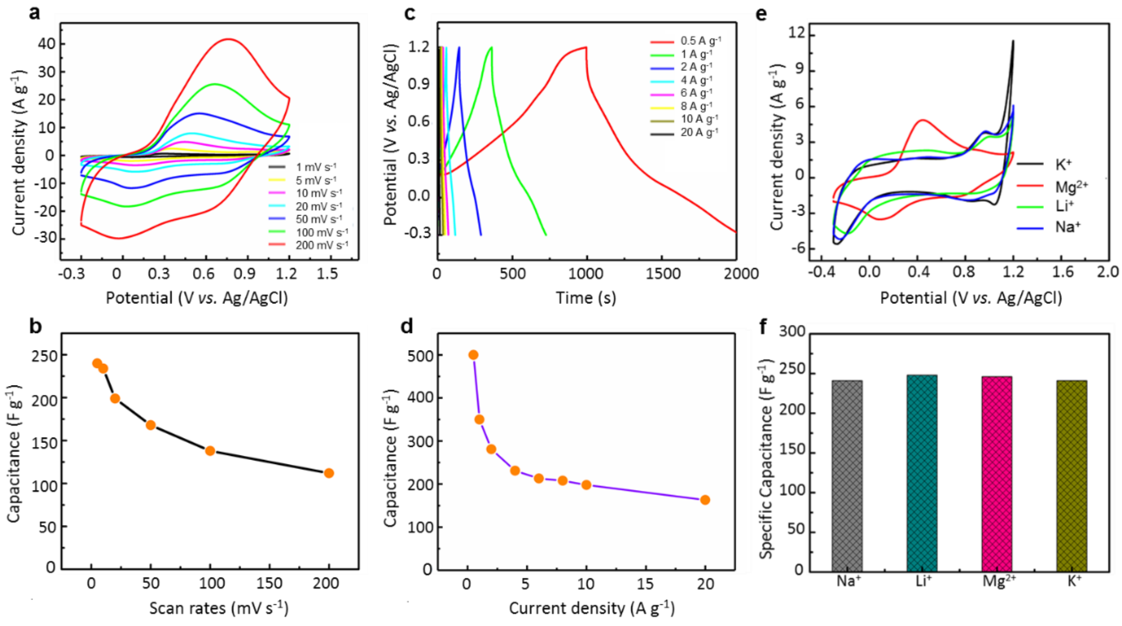

Supplementary Figure 5. Electrochemical performances of MnO_2@C in a three-electrode system. (a) CV curves of an MnO_2@C electrode in 1.0 M $MgSO_4$ at different scan rates from 1 to 200 mV s^{-1}. (b) Corresponding specific capacitances at different scan rates. (c) GCD curves of an MnO_2@C electrode in 1.0 M $MgSO_4$ at different current densities from 0.5 to 20 A g^{-1}. (d) Corresponding specific capacitances at different current densities. (e) CV curves at a scan rate of 10 mV s^{-1} in different neutral electrolytes. (f) Corresponding specific capacitances in different neutral electrolytes.

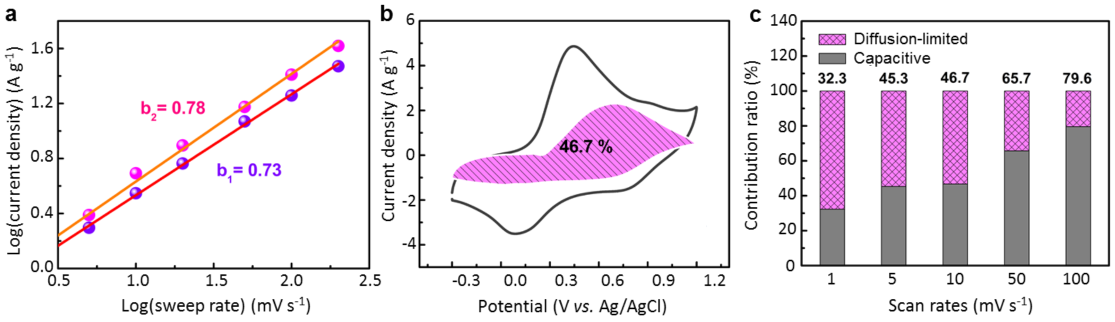

Supplementary Figure 6. *b*-value determination and capacitive contribution of MnO_2@C in 1.0 M $MgSO_4$. (a) *b*-value of the anodic peak and cathodic peak of an MnO_2@C electrode. (b) Capacitive and diffusion controlled contributions to the total charge storage of an MnO_2@C composite at 10 mV s^{-1}. (c) Separation of contributions from capacitive and diffusion ratio at different scan rates.

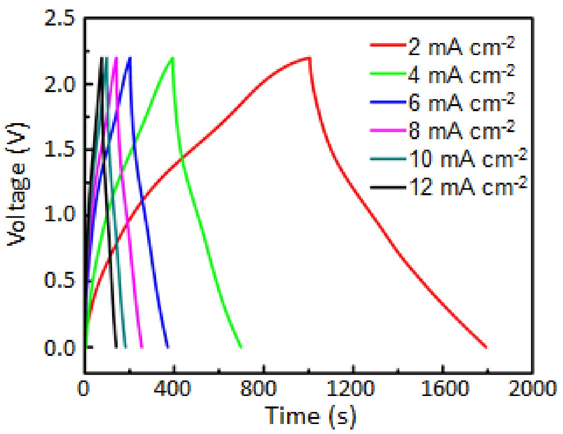

Supplementary Figure 7. GCD curves of a liquid electrolyte based ASC device at different current densities.

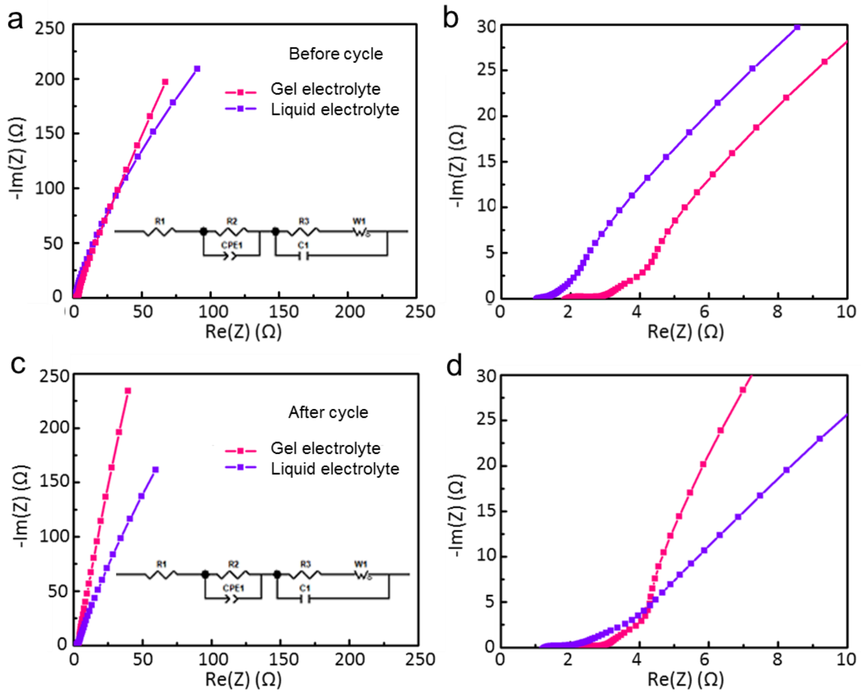

Supplementary Figure 8. Comparative EIS profiles in a liquid electrolyte and a gel electrolyte. (a) EIS spectra of the gel electrolyte and liquid electrolyte based systems before cycle test. (b) Detailed high-frequency region corresponding to (a). (c) EIS spectra of the gel electrolyte and liquid electrolyte based systems after cycle test. (d) Detailed high-frequency region corresponding to (c). The inset graph in (a) and (c) is a Randles equivalent circuit.

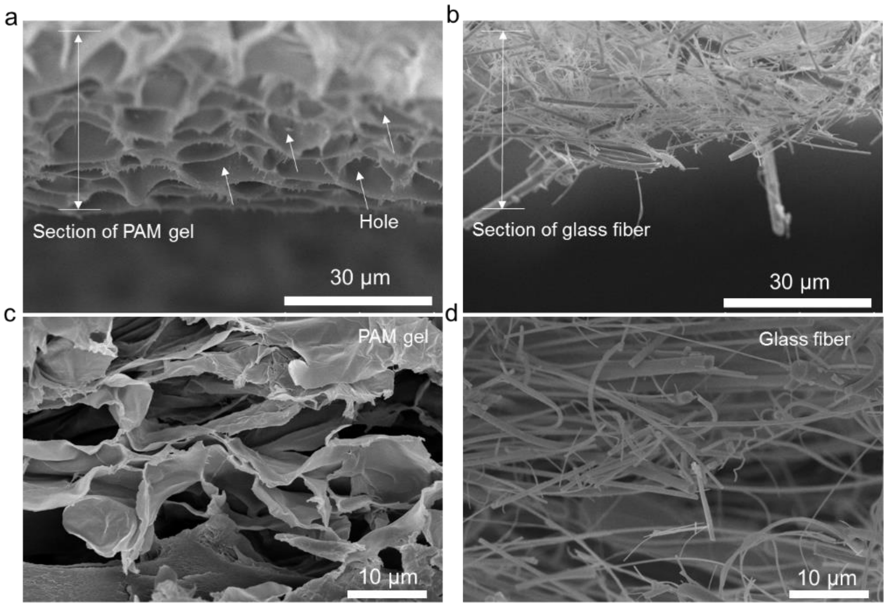

Supplementary Figure 9. Morphological characterization of a gel- and liquid-electrolyte based ASC device. (a, b) Cross-sectional SEM images of the (a) PAM gel and (b) glass fiber separator (liquid electrolyte system). (c, d) Top-view SEM images of the (c) PAM gel and (d) glass fiber separator (liquid electrolyte system).

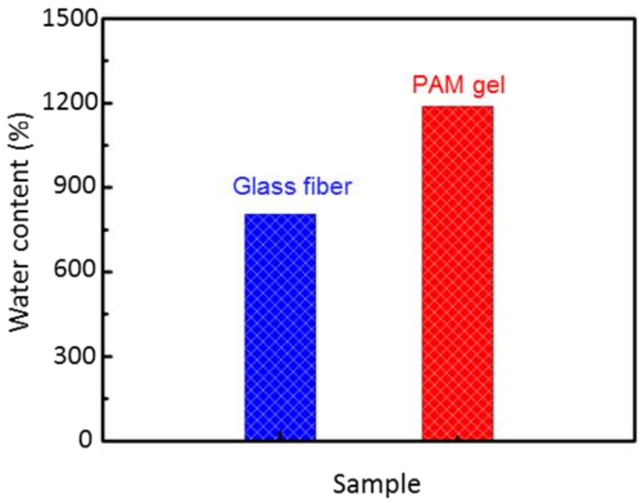

Supplementary Figure 10. Comparison of the water contents under identical testing conditions of the glass fiber separator and the PAM gel.

a b

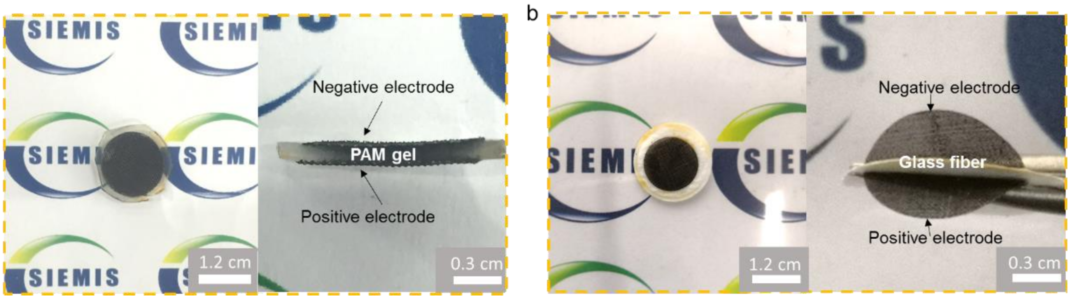

Supplementary Figure 11. Comparison between the real photos of ASCs with the gel electrolyte and the liquid electrolyte. (a) Digital photos of the interface of gel electrolyte (top view and cross-sectional view). (b) Digital photos of the interface of glass fiber separator using liquid electrolyte (top view and cross-sectional view).

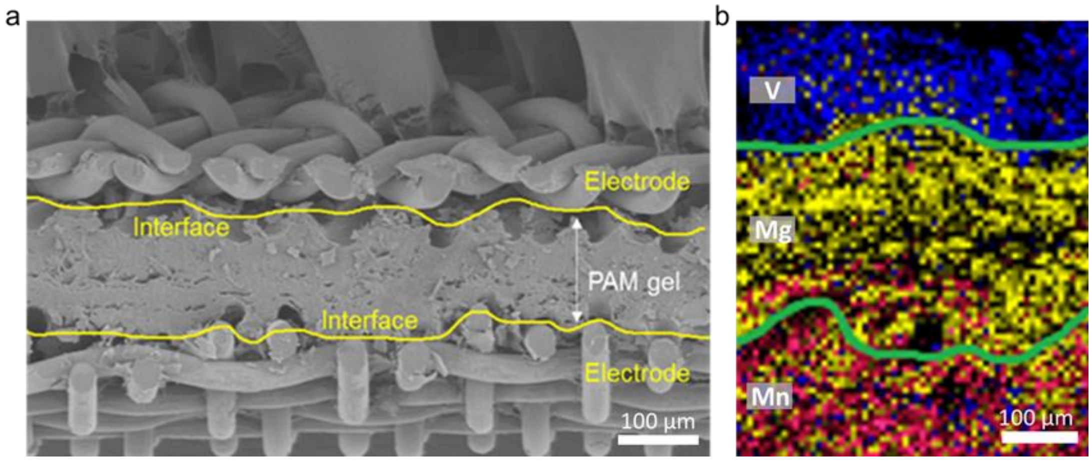

Supplementary Figure 12. Tight interfacial contact in a gel electrolyte system. (a) SEM image of the interface between electrodes and gel. (b) Elemental mapping of V, Mg and Mn at the interface.

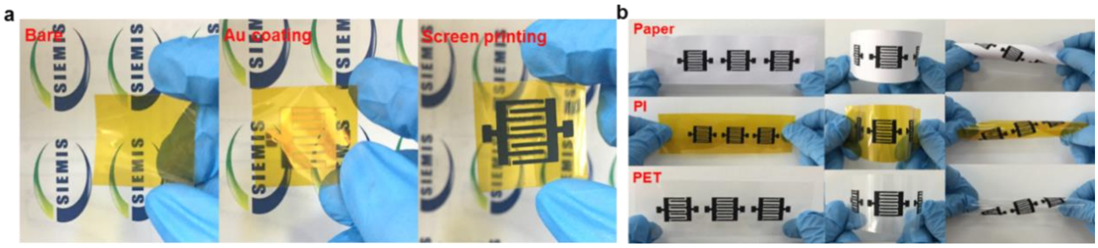

Supplementary Figure 13. Digital photos of printing-derived flexible MASCs. (a) Photos of the fabricated process of MASCs. (b) MASCs printed on different substrates (PI, PET and paper), showing great flexibility.

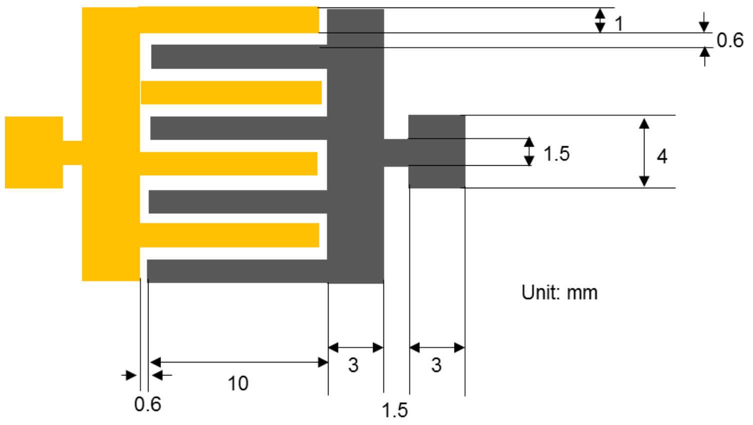

Supplementary Figure 14. Detailed dimensions of the screen-printing pattern.

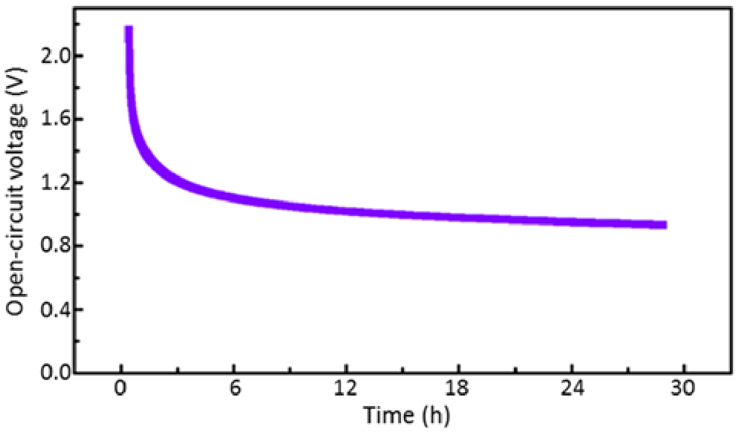

Supplementary Figure 15. The self-discharge curves of the MASCs. The self-discharge performance of printed MASCs have been probed by recording the open circuit voltage from the maximum 2.2 V to 1.0 V. It exhibits a self-discharge performance of 12 h to maintain 1.1 V (half the maximum voltage) and 18 h to maintain 1.0 V, outperforming most of the other results reported to date.

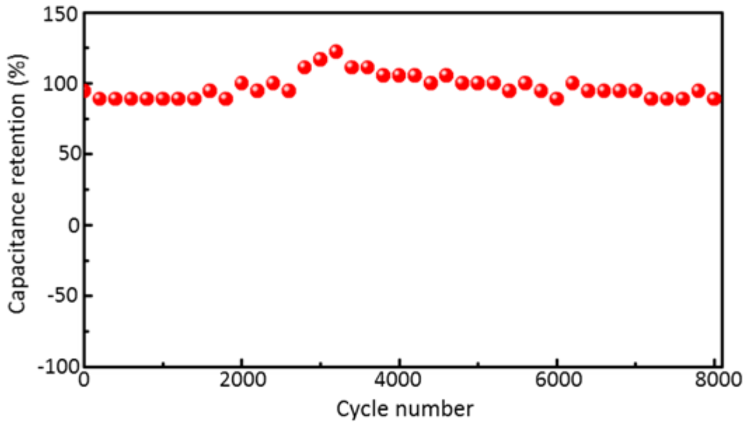

Supplementary Figure 16. Long-term cycling stability of a MASC at a charge/discharge current density of 2 mA cm^{-2}.

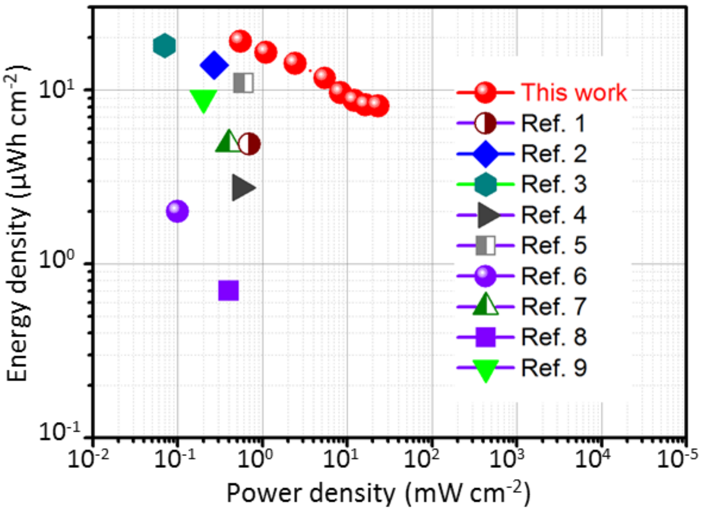

Supplementary Figure 17. Ragone plot of the printing-derived flexible MASCs. Ragone plot of the MnO_2@C//VN micro-supercapacitor in comparison with other recently reported micro-supercapacitors.

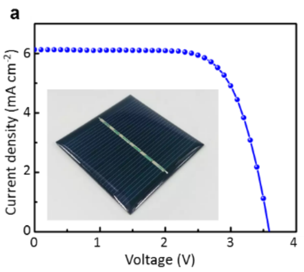

b

V_{oc}/V	I_{sc}/mA	P_{max}/mW	V_{pmax}/V
3.5	149.96	378.37	2.8

I_{pmax}/mA	FF	η/%
135.13	72.09	15.46

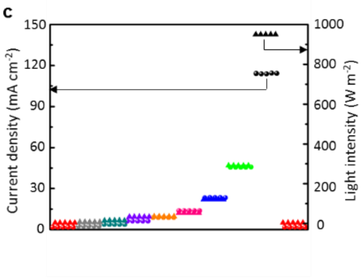

Supplementary Figure 18. Photoelectric performance of a polycrystalline Si solar cell. (a) J-V curve. Inset: Digital photo showing the device. (b) Detailed parameter indicator of the solar cell. (c) Detailed information on light intensity and the corresponding output current.

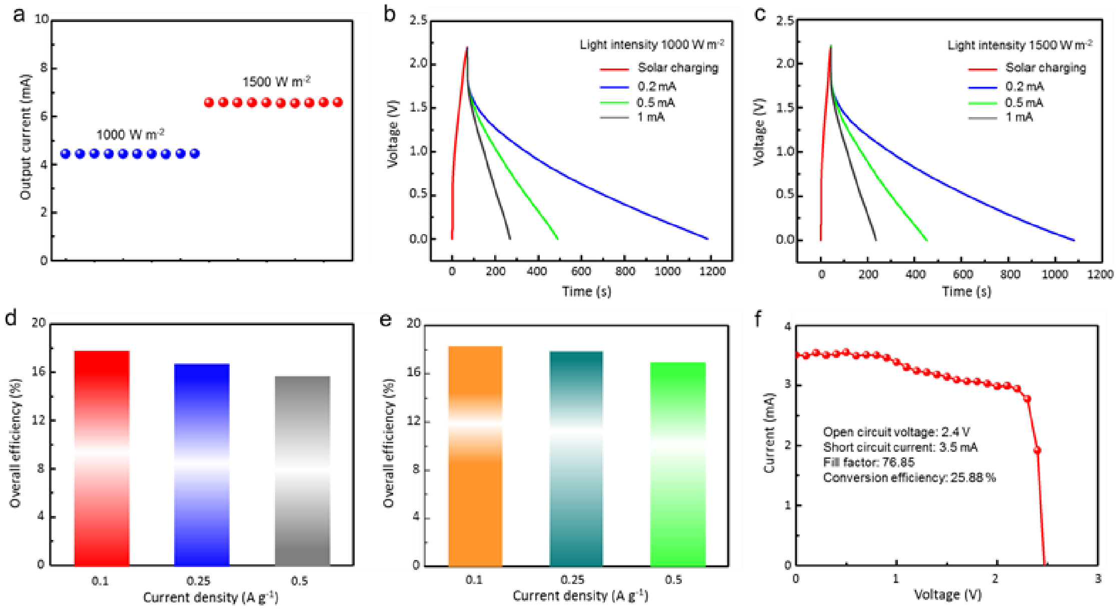

Supplementary Figure 19. Photoelectric performance of a GaAs solar cell. (a) Response photocurrent of the GaAs solar cell at different light intensities. (b) Voltage-time profiles of our solar-charging integrated units with different discharge current under 1000 W m^{-2}. (c) Voltage-time profiles of our solar-charging integrated units with different discharge current under 1500 W m^{-2}. (d, e) The overall efficiency calculated according to (b) and (c) at different discharge current values. (f) J-V curve of the GaAs solar cell.

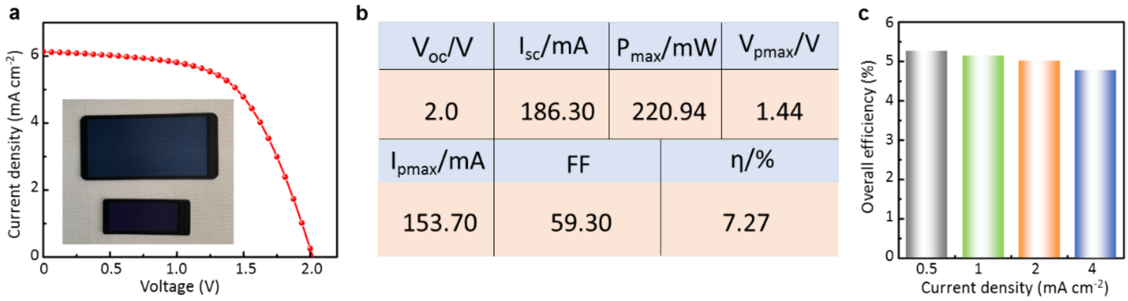

V_{oc}/V	I_{sc}/mA	P_{max}/mW	V_{pmax}/V
2.0	186.30	220.94	1.44
I_{pmax}/mA	FF	η/%	
153.70	59.30	7.27	

Supplementary Figure 20. Photoelectric performance of a flexible amorphous Si solar cell. (a) J-V curve. Inset: Digital photo showing the device. (b) Detailed parameter indicator of the solar cell. (c) Calculated overall efficiency of our flexible solar-charging units (derived from the flexible Si solar cell) at different discharge current densities from 0.5 to 4 mA cm^{-2}.

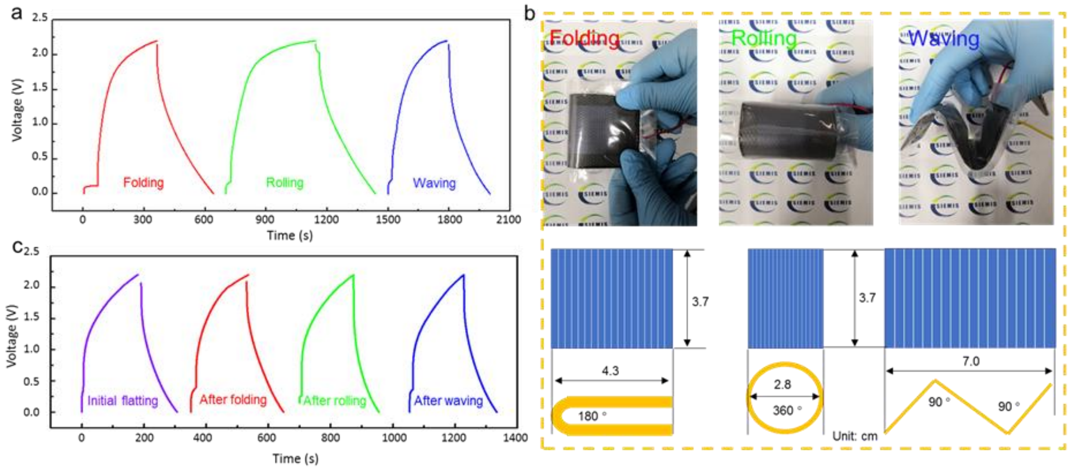

Supplementary Figure 21. Flexibility tests of the integrated unit. (a) Solar-charging and electrical discharging curves under different flexible conditions (folding, rolling and waving). (b) Digital photos and detailed parameter of the integrated unit in three different deformed characterizations. (c) Solar charging and electrical discharging curves (initial state, after folding, after rolling and after waving).

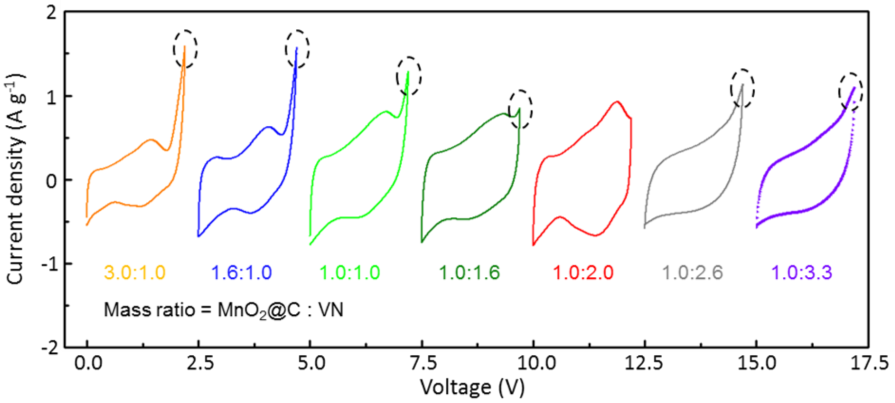

Supplementary Figure 22. CV curves with different mass ratios of positive and negative electrodes at a scan rate of 10 mV s^{-1}.

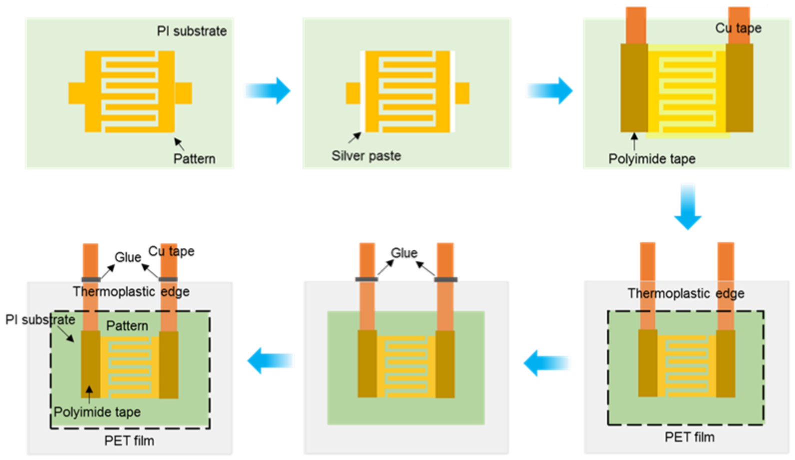

Supplementary Figure 23. The packaging process of the printable MASC.

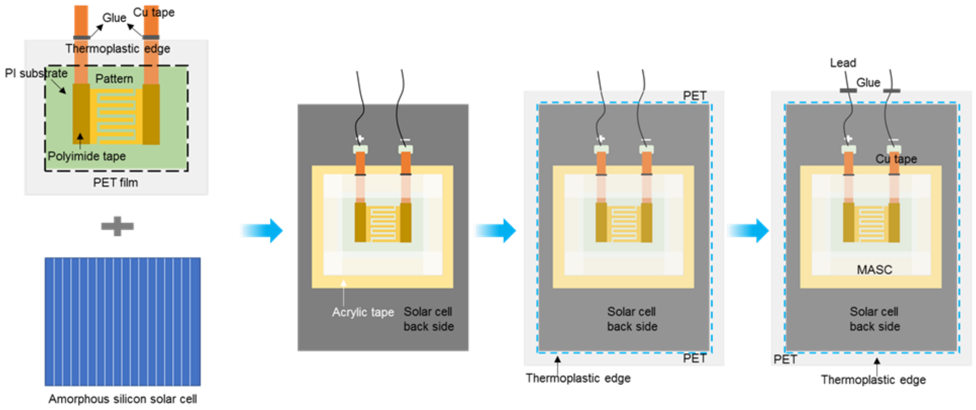

Supplementary Figure 24. The packaging process of the flexible solar charging unit.

Supplementary Tables

Supplementary Table 1. Equivalent Circuit Parameters Obtained from the Fitting Results for Components of the Equivalent Circuit Fit with the EIS Spectra

E_{chem} systems	R_s (Ω)	R_{ct} (Ω)	CPE-T (mF/cm^2)	CPE-P	A (Ω S^{-n})	*n*	C_F	R_{leak} (Ω)
VN in Li_2SO_4	4.22	0.79	3.04	0.90	1.02	0.43	0.75	0.82
VN in Na_2SO_4	6.92	1.56	1.28	0.84	1.16	0.45	0.25	0.51
VN in K_2SO_4	8.54	1.49	1.92	0.81	3.70	0.44	0.22	0.49
VN in $MgSO_4$	5.46	1.2	1.48	0.90	0.91	0.45	0.34	0.47

Supplementary Table 2. Equivalent Circuit Parameters Obtained from the Fitting Results for Components of the Equivalent Circuit Fit with the EIS Spectra

Electrolyte type	R_1 (Ω)	R_2 (Ω)	W_1-P
PAM gel (before cycle)	1.981	0.852	0.406
PAM gel (after cycle)	2.018	0.895	0.434
Liquid (before cycle)	1.118	0.380	0.360
Liquid (after cycle)	1.361	0.785	0.396

Supplementary Table 3. Performance Comparisons of Recently Reported In-plane Supercapacitor Devices

System	Electrolyte (gel)	Voltage (V)	Energy (μWh/cm^2)	Power (mW/cm^2)	Ref.
MnO_2@C//VN	$MgSO_4$/PAM	0-2.2	19.13	22.96	This work
3D Porous C	LiTFSI	0-2.5	4.9	7.92	1
NiCoP@NiOOH//ZIF	KOH/PVA	0-1.4	13.9	2	2
$Cu(OH)_2$@FeOOH/Cu	[EMIM][BF_4]/SiO_2	0-1.5	18.07	0.7	3
3DGN/SWNT/AgNW	LiCl/PVA	0-1.0	2.75	0.361	4
Ppy@MWCNT//MnO_2@Ppy	LiCl/PVA	0-1.6	12.16	17.65	5
MoS_2@rGO-CNT	H_2SO_4/PVA	0-1.0	2	1	6
3D graphene	H_2SO_4/PVA	0-1.0	0.38	14.4	7
$Ti_3C_2T_x$	H_2SO_4/PVA	0-0.5	0.32	0.158	8
MnO_2//PPy	CMC-Na_2SO_4	0-1.5	8.05	7.32	9

Supplementary Table 4. Related Parameters of the Rigid Solar-charging Self-powered System with Discrete Designs

Conversion system	Storage system	Overall efficiency	Conversion efficiency	Storage efficiency	Output voltage (V)	Endurance current (max)	Ref.
GaAs	ASC	17.57 %	25.88 %	67.90 %	2.20	114 mA cm^{-2}	This
p-Si	ASC	11.95 %	15.46 %	77.31 %	2.20	114 mA cm^{-2}	work
GaAs	FB	14.10 %	26.10 %	54.02 %	1.25	50 mA cm^{-2}	10
PSC	LIB	9.36 %	14.40 %	69.44 %	3.14	4 C	11
DSC	LIB	5.50 %	7.89 %	69.71 %	3.14	4 C	11
PSC	LIC	8.41 %	15.00 %	56.07 %	3.00	5 A g^{-1}	12
PSC	LIB	7.80 %	12.65 %	61.66 %	2.60	2 C	13
PSC	SSC	10.00 %	13.60 %	73.53 %	1.45	15 mA cm^{-2}	14
PSC	SSC	5.10 %	6.10 %	83.61 %	1.20	N/A	15
OSC	SSC	2.92 %	5.20 %	56.15 %	0.53	10 mA cm^{-2}	16

Annotation: PSC: perovskite solar cell; DSC: dye sensitized solar cell; p-Si: polysilicon; c-Si: crystalline silicon; OSC: organic solar battery; ASC: asymmetric supercapacitor; SSC: symmetric supercapacitor; FB: flow battery; LIB: Li ion battery; LIB: Li ion capacitor.

Supplementary Table 5. Related Parameters of the Flexible Solar-charging Self-powered Systems

System	Structure	$\eta_{(overall)}$	Output voltage	Cycle number	Ref.
a-Si//MASC	In plane	5.20 %	2.20 V	100 (98.70 %)	This work
DSC//SSC	In plane	N/A	1.10 V	10 (54.55 %)	17
DSC//SSC	Fiber	0.90 %	1.20 V	N/A	18
DSC//SSC	In plane	N/A	1.80 V	10	19
SC//ASC	Fiber	N/A	1.55 V	N/A	20
a-Si//SSC	In plane	N/A	0.85 V	N/A	21
DSC//SSC	Fiber	1.80 %	0.70 V	5	22
PSC//ASC	In plane	4.90 %	0.60 V	10	23
PC//SSC	Fiber	0.85 %	0.60 V	1000	24

Annotation: PSC: perovskite solar cell; DSC: dye sensitized solar cell; a-Si: amorphous silicon; PC: polymer solar battery; MASC: micro-asymmetric supercapacitor; ASC: asymmetric supercapacitor; SSC: symmetric supercapacitor.

Supplementary Notes

Supplementary Note 1.

The measured Nyquist plots were well fit based on an equivalent Randles circuit by using the following equation:

$$C = R_s + \frac{1}{j\omega CPE + \frac{1}{R_{ct} + W_o}} + \frac{1}{j\omega C_l + \frac{1}{R_{leak}}}$$

where R_s is the internal resistance of the cell, CPE stands for the constant phase element (CPE), R_{ct} is the charge transfer resistance, W_o is the Warburg element, C_l is the low frequency mass capacitance, and R_{leak} is the low frequency leakage resistance.

For a supercapacitor, especially the current system including the pseudocapacitive processes, the fit capacitive component in the EIS profile might not behave ideally, but act like a CPE instead. Regarding these EIS results for the VN electrodes in different aqueous cation systems, the Nyquist curves are better interpreted when the CPE is replaced by the pure capacitance C_{dl}. The CPE component can be fit into two key parameters, *i.e.*, CPE-T and CPE-P according to the following equation:

$$Z_{CPE} = \frac{1}{T_0 (j\omega)^n}$$

CPE-T is related to the pseudocapacitance and CPE-P is related to the semi-circle in the Nyquist plot (depressed semicircle), normally used for the notation '*n*' in the equation. The more approximate to 1 the CPE-P parameter is the more ideal is the capacitive behavior of the electrochemical system.

Other parameters were also fit according to the non-linear calculation by defining different Randles circuit components. In the high frequency region, the point of intersection on the real axis represents the internal resistance R_s, which includes the intrinsic electronic resistance of the electrode material, the ohmic resistance of the electrolyte, and the interfacial resistance between the electrode and the current collector. The semicircle in the high frequency region provides the behavior of the interfacial charge transfer resistance R_{ct}. The Nyquist plot exhibits a straight long tail (that seems almost perpendicular to the *x*-axis) and stretches to the low frequency region. This line represents the mass capacitance C_l, and the inclined angle suggests a resistive element, which is the leakage resistance R_{leak}. The transmission line with an angle of nearly 45 degrees to the x-axis from the high frequency to the mid-frequency represents the Warburg element W_o, which is expressed as:

$$W_o = \frac{A}{(j\omega)^n}$$

where *A* is the Warburg coefficient, ω is the angular frequency, and *n* is an exponent.

Supplementary Note 2.

The mass ratio of the positive to negative electrode is obtained by adopting the following equation:

$$\frac{m+}{m-} = \frac{C-\Delta V-}{C+\Delta V+}$$

where m+ and m− are the mass loading of the MnO_2@C and VN electrodes, respectively. C+ and ΔV+ are the specific capacitance and the potential window of the MnO_2@C electrode, respectively. C− and ΔV− are the specific capacitance and the potential window of the VN electrode, respectively.

Based on three-electrode electrochemical measurements and the CV curve shwon in Fig. 4B, the VN electrode exhibits a specific capacitance of 230 F g^{-1} with a potential window of 0.9 V, while the MnO_2@C electrode shows a specific capacitance of 240 F g^{-1} with a broad potential window of 1.5 V. According to the loading mass balance calculation formula, the theoretical mass ratio of the positive:negative electrode is 1:1.74. To optimize the positive/negative electrode mass loading ratio, we have also tested the CV curves with different mass ratios as summarized in Supplementary Fig. 17. It is clear that the asymmetric supercapacitor with a mass ratio of 1.0:2.0 exhibits the widest stable working voltage of 2.2 V without any obvious gas-evolution-induced electrochemical polarization. As for the device with a mass ratio of 1.0:1.6, it also manifests an operating voltage of 2.2 V, but with the presence of an oxygen evolution reaction related peak. Here it is worth mentioning that we are not able to achieve precise control on a mass loading ratio between 1.0:1.6 and 1.0:2.0, hence, we decided to fabricate the quasi-solid-state ASC and printed MASC with a mass loading (mg cm^{-2}) ratio approaching ~1.0:2.0. For the screen-printing process, we control the single printing of the MnO_2@C positive electrode with a mass loading of 0.98 mg cm^{-2} and the VN negative electrode with a mass loading of 0.88 mg cm^{-2} (by comparing the mass difference before and after screen printing). Therefore, we simply screen printed the VN negative electrode twice and the MnO_2@C positive electrode once for the final printed MASC device with a final mass loading ratio of 1.0:1.76 (the positive and negative electrode loading masses are 0.98 and 1.72 mg cm^{-2}, respectively).

Supplementary References

1 Ma, X. *et al.* High energy density micro-supercapacitor based on a three-dimensional bicontinuous porous carbon with interconnected hierarchical pores. *ACS Appl. Mater. Interfaces* **11**, 948-956 (2019).

2 Qiu, M., Sun, P., Cui, G., Tong, Y. & Mai, W. A flexible microsupercapacitor with integral photocatalytic fuel cell for self-charging. *ACS Nano* **13**, 8246-8255 (2019).

3 Xie, J.-Q. *et al.* In situ growth of $Cu(OH)_2$@FeOOH nanotube arrays on catalytically deposited Cu current collector patterns for high-performance flexible in-plane micro-sized energy storage devices. *Energy Environ. Sci.* **12**, 194-205 (2019).

4 Kim, S.-W. *et al.* Plotter-assisted integration of wearable all-solid-state micro-supercapacitors. *Nano Energy* **50**, 410-416 (2018).

5 Gao, J. *et al.* Laser-assisted large-scale fabrication of all-solid-state asymmetrical micro-supercapacitor array. *Small* **14**, 1801809 (2018).

6 Yang, W. *et al.* Carbon-MEMS-based alternating stacked MoS_2@rGO-CNT micro-supercapacitor with high capacitance and energy density. *Small* **13**, 1700639 (2017).

7 Zhang, L. *et al.* Flexible micro-supercapacitor based on graphene with 3D structure. *Small* **13**, 1603114 (2017).

8 Zhang, C. *et al.* Additive-free MXene inks and direct printing of micro-supercapacitors. *Nat. Commun.* **10**, 1795 (2019).

9 Guo, R. *et al.* In-plane micro-supercapacitors for an integrated device on one piece of paper. *Adv. Funct. Mater.* **27**, 1702394 (2017).

10 Li, W. *et al.* 14.1% Efficient monolithically integrated solar flow battery. *Chem* **4**, 2644-2657 (2018).

11 Gurung, A. *et al.* Highly efficient perovskite solar cell photocharging of lithium ion battery using DC-DC booster. *Adv. Energy Mater.* **7**, 1602105 (2017).

12 Li, C. *et al.* Flexible perovskite solar cell-driven photo-rechargeable lithium-ion capacitor for self-powered wearable strain sensors. *Nano Energy* **60**, 247-256 (2019).

13 Xu, J. *et al.* Efficiently photo-charging lithium-ion battery by perovskite solar cell. *Nat. Commun.* **6**, 8103 (2015).

14 Xu, X. *et al.* A power pack based on organometallic perovskite solar cell and supercapacitor. *ACS Nano* **9**. 1782-1787 (2015).

15 Liang, J. *et al.* An all-inorganic perovskite solar capacitor for efficient and stable spontaneous photocharging. *Nano Energy* **52**, 239-245 (2018).

16 Lechêne, B. P. *et al.* Organic solar cells and fully printed super-capacitors optimized for indoor light energy harvesting. *Nano Energy* **26**, 631-640 (2016).

17 Cai, J. *et al.* A. High-performance all-solid-state flexible carbon/TiO_2 micro-supercapacitors with photo-rechargeable capability. *RSC Adv.* **7**, 415-422 (2017).

18 Chai, Z. *et al.* Tailorable and wearable textile devices for solar energy harvesting and simultaneous storage. *ACS Nano* **10**, 9201-9207 (2016).

19 Dong, P. *et al.* A flexible solar cell/supercapacitor integrated energy device. *Nano Energy* **42**, 181-186 (2017).

20 Gao, Z. *et al.* Cotton-textile-enabled flexible self-sustaining power packs *via* roll-to-roll fabrication. *Nat. Commun.* **7**, 11586 (2016).

21 Manjakkal, L. *et al.* Flexible self-charging supercapacitor based on graphene-Ag-3D graphene foam electrodes. *Nano Energy* **51**, 604-612 (2018).

22 Liang, J. *et al.* MoS_2-based all-purpose fibrous electrode and self-powering energy fiber for efficient energy harvesting and storage. *Adv. Energy Mater.* **7**, 1601208 (2017).

23 Zhang, F. *et al.* Highly flexible and scalable photo-rechargeable power unit based on symmetrical nanotube arrays. *Nano Energy* **46**, 168-175 (2018).

24 Zhang, Z. *et al.* Integrated polymer solar cell and electrochemical supercapacitor in a flexible and stable fiber format. *Adv. Mater.* **26**, 466-470 (2014).
